# Biphasic ROS production, p53 and BIK dictate the mode of cell death in response to DNA damage in colon cancer cells

**DOI:** 10.1371/journal.pone.0182809

**Published:** 2017-08-10

**Authors:** Ozgur Kutuk, Nurgul Aytan, Bahriye Karakas, Asli Giray Kurt, Ufuk Acikbas, Sehime Gulsun Temel, Huveyda Basaga

**Affiliations:** 1 Department of Medical Genetics, Baskent University School of Medicine, Adana Dr. Turgut Noyan Medical and Research Center, Adana, Turkey; 2 Department of Neurology, Boston University School of Medicine, Boston, United States of America; 3 Molecular Biology, Genetics and Bioengineering Program, Sabanci University, Istanbul, Turkey; 4 Department of Histology and Embryology, Uludag University School of Medicine, Bursa, Turkey; 5 Department of Histology and Embryology, Near East University School of Medicine, Nicosia, Northern Cyprus; Institute of Biochemistry and Biotechnology, TAIWAN

## Abstract

Necrosis, apoptosis and autophagic cell death are the main cell death pathways in multicellular organisms, all with distinct and overlapping cellular and biochemical features. DNA damage may trigger different types of cell death in cancer cells but the molecular events governing the mode of cell death remain elusive. Here we showed that increased BH3-only protein BIK levels promoted cisplatin- and UV-induced mitochondrial apoptosis and biphasic ROS production in HCT-116 wild-type cells. Nonetheless, early single peak of ROS formation along with lysosomal membrane permeabilization and cathepsin activation regulated cisplatin- and UV-induced necrosis in p53-null HCT-116 cells. Of note, necrotic cell death in p53-null HCT-116 cells did not depend on BIK, mitochondrial outer membrane permeabilization or caspase activation. These data demonstrate how cancer cells with different p53 background respond to DNA-damaging agents by integrating distinct cell signaling pathways dictating the mode of cell death.

## Introduction

The mitochondrial apoptotic pathway is strictly regulated by the selective protein-protein interactions between antiapoptotic and proapoptotic BCL-2 protein family members[1]. In response to various cellular stresses, activation of BAX and BAK by activator BH3-only proteins (BIM, BID and PUMA) coincide with the suppression of antiapoptotic BCL-2 proteins (BCL-2, BCL-XL, MCL-1) by sensitizer BH3 proteins (BAD, BIK, NOXA, BMF, HRK) and is followed by the permeabilization of the mitochondrial outer membrane. Mitochondrial outer membrane permeabilization and the release of cytochrome *c* into the cytosol represent the point of no return for the commitment of cell death. In the cytosol, cytochrome c, APAF-1 and caspase-9 form the apoptosome complex and activated caspase-9 triggers the activation of executioner caspase-3 and caspase-7 by cleaving them.

BH3-only protein BIK has been shown to act as a tumor suppressor and deletions in 22q13.2 and 22q13.3 chromosomal regions containing the *Bik* locushave been reported in colorectal cancers, head and neck cancers, gliomas and renal cell carcinomas[2]. Identified as the founding member of the BH3-only proteins interacting with BCL-2 and BCL-XL, BIK contains an already-exposed and highly conserved BH3 domain and C-terminal domain, both required for maximal cell death activity[2–4]. BIK was also demonstrated to mediate cell death in response to chemotherapeutics and cellular stress in various epithelial and non-epithelial cancer cell lines[5–10]. Furthermore, BIK expression was shown to be tightly regulated at transcriptional, translational and post-translational levels, with p53, E2F, STAT1 and Smad3/4 among the candidate transcriptional factors[2].

The level of intracellular reactive oxygen species (ROS) is regulated by pro-oxidant and antioxidant mechanisms. Intracellular ROS such as superoxide, hydroxyl radical and hydrogen peroxide are the main byproducts of mitochondrial respiration and can either act as cell-damaging molecules or as secondary messengers depending on the amplitude and duration of ROS formation. DNA damaging agents have been shown to increase ROS levels, regulating cell death, metabolism and proliferation[11–14]. These effects of DNA damage-induced ROS formation can be coupled to p53 transcriptional activity, ROS and p53 regulating each other reciprocally in different experimental settings[15–18]. In addition, DNA damage-induced cell death was demonstrated to proceed throughp53-dependent and -independent signaling pathways[16, 19–21]. On the other hand, cathepsin activation and lysosomal membrane permeabilization were shown to mediate cell death in response to various cellular stress inducers including DNA damage by using p53-dependent or -independent pathways[17, 22–26]. Increased lipid peroxidation, destabilization of lysosomal membranes and oxidation of lysosomal membrane proteins contribute to ROS-mediated lysosomal leakage.

In this study, we aimed to determine how DNA damage-induced cell death signals are differentially transduced in p53-wt and p53-null colon cancer cells by BH3-only protein BIK and reactive oxygen species. Our data demonstrate that BIK is involved in the DNA damage-induced mitochondrial apoptosis pathway and biphasic ROS production in HCT-116 wt cells. In contrast, increased ROS formation along with lysosomal membrane permeabilization and cathepsin B/L activation mediates necrotic cell death in HCT-116 p53 -/- cells, which does not depend on BIK, mitochondrial outer membrane permeabilization or caspase activation.

## Materials and methods

### Cell lines

HCT-116 wt and HCT-116 p53 -/- cells were kindly provided by Bert Vogelstein (Howard Hughes Medical Institute, Johns Hopkins University, USA). Cells were grown in McCoy’s 5A medium (ThermoFisher Scientific) supplemented with 10% heat-inactivated fetal bovine serum (Sigma, St Louis, MO, USA), 100 IU/ml penicillin, and 100 mg/ml streptomycin (Thermo Scientific) in a humidified incubator at 37°C and 5% CO_2_. For 3D cell cultures, cell spheroids were grown in AlgiMatrix 24-well plates (ThermoFisher Scientific) as recommended by the manufacturer. Spheroids were isolated from matrix using cell dissociation buffer (ThermoFisher Scientific) for lysis and protein isolation. Stratalinker 2400 (254 nm UVC source, Stratagene, La Jolla, CA) was used for UV-irradiation.

### Chemicals

Cisplatin, trehalose, oligomycin, digitonin, succinate, FCCP, TIRON, NAC (N-Acetyl-L-cysteine), 4-ANI (4-Amino-1,8-naphthalimide), Necrostatin-1, Q-VD-OPh CA-074Me and cycloheximide were purchased from Sigma. BSA and Z-FA-FMK was obtained from Enzo Life Sciences.

### Cell viability, cell death and apoptotic assays

Cell viability was determined in HCT-116 wt and HCT-116 p53 -/- cells by CellTiterGlo assay according to the manufacturer’s protocol (Promega). Results are expressed as % of untreated control (mean±SEM). Apoptosis was evaluated as levels of specifically DEVDase-cleaved CK-18 in total cell lysates by using a M30 Apoptosense assay (PEVIVA AB) and total cell death (apoptosis+necrosis) was evaluated by a M65 Apoptosense assay (PEVIVA AB) as described before[27]. Results were presented as fold increase of untreated control (mean±SEM). alamarBlue assay (ThermoFisher Scientific) was used to assess cell viability in cell spheroids grown in 3D culture as described by the manufacturer.

### Coimmunoprecipitation and immunoblotting

Total cell lysates were prepared in 1% CHAPS buffer [5mM MgCl2, 140 mMNaCl, 1mM EDTA, 1mM EGTA, 1% CHAPS, 20mM Tris-HCl (pH 7.5), and protease inhibitors (cOmplete ULTRA, Roche)]. AlgiMatrix dissolving buffer (ThermoFisher Scientific) was used to harvest spheroids before lysis in 1% CHAPS buffer. Proteins (600–1000 μg) were immunoprecipitated with BCL-2 (#4223, Cell Signaling), BCL-XL (#2762, Cell Signaling), MCL-1 (S-19, Santa Cruz) antibodies at 4°C for 16h and coimmunoprecipitates were captured by Dynabeads Protein G at 4°C for 2 h. Beads were recovered using DynaMag spin magnet and washed twice in 1% CHAPS buffer. Total cell lysates and immunoprecipitates were separated on NuPage 10% Bis-Tris gels. After SDS-PAGE, proteins were transferred onto PVDF membranes (Millipore) and then blocked with 5% dried milk in PBS-Tween20. Membranes were incubated with primary and secondary antibodies (GE Healthcare) in a buffer containing 10% milk diluent-blocking concentrate (KPL), detected with Luminata Crescendo Western HRP substrate (Millipore). Blots were imaged with LAS4000 image analyzer (Fujifilm) on chemiluminescence mode. The following antibodies were used for immunoblotting: BCL-2 (#2872, Cell Signaling), BCL-XL (#2762, Cell Signaling), Actin (#8457, Cell Signaling), BIK (#4592, Cell Signaling), MCL-1 (S-19, Santa Cruz), p53 (DO-1, Santa Cruz).

### BH3 profiling

JC-1 (ThermoFisher Scientific) plate-based BH3 profiling was done as described before[28, 29]. GeneCust Europe synthesized the peptides used in this assay and peptide sequences were previously described[29]. Briefly, cells were permeabilized in T-EB buffer [300 mM Trehalose, 10 mM Hepes-KOH, pH 7.7, 80 mM KCl, 1 mM EGTA, 1 mM EDTA, 0.1% BSA (w/v), 5mM succinate] in the presence of 0.005% digitonin, 5 mM β-mercaptoethanol, 10 μg/ml oligomycin, 1 μM JC-1. Cells were transferred to 364-well black plates and treated with BH3 peptides (100 μM). JC-1 fluorescence was analyzed at 545 nm excitation and 590 nm emission using Spectramax Gemini multiplate fluorometer every 5 min for 3h at 28–32°C. Data shown are mean±SEM of three independent experiments in duplicate and expressed as %ΔΨm (%MMP) loss compared with DMSO-treated cells. FCCP was used as a positive control. Cytochrome c ELISA-based BH3 profiling was carried out as described before[29].

### siRNA transfection

Cells were transfected with BIK siRNA (Hs_BIK_5 FlexiTube siRNA, NM_001197, Qiagen) and negative control (scrambled) siRNA (AllStars Negative Control siRNA, Qiagen) by using Hiperfect transfection reagent (Qiagen) according to manufacturer’s instructions. Protein knockdown efficiencies by siRNA transfection were verified by immunoblotting 24h following transfection. For 3D cell culture experiments, cells were transfected with corresponding siRNA duplex for 12h and transferred to AlgiMatrix 3D culture plate for the growth of cell spheroids in the presence of siRNA treatment for every 24h. Protein knockdown efficiencies in cell spheroids were verified by immunoblotting.

### Caspase activation assays

The activity of caspase-3 and caspase-9 was determined by ApoAlert Caspase Profiling Plate (Clontech) according to manufacturer’s protocol. The release of fluorochrome AMC was analyzed at 380 nm excitation and 460 nm emission using Spectramax Gemini microplate fluorometer. Data shown are mean±SEM of three independent experiments and expressed in arbitrary fluorescence units per mg of protein.

### Galectin-3 staining

Galectin-3 immunofluorescence staining to evaluate lysosomal membrane permeabilization was performed as described before[30]. Briefly, cells were grown in Millicell EZ SLIDE 4-well glass chamber slides in McCoy’s 5A medium (ThermoFisher Scientific) supplemented with 10% heat-inactivated fetal bovine serum (Sigma, St Louis, MO, USA), 100 IU/ml penicillin, and 100 mg/ml streptomycin (Thermo Scientific) in a humidified incubator at 37°C and 5% CO_2_.?? and subjected to treatments as indicated. Cells were fixedin 4%paraformaldehyde for 10 min at room temperature, washed once in cold PBS and incubated for 10 min in ammonium chloride solution. Cells were washed twice in cold PBS and then permeabilized using 1% BSA, 0.3% Triton X-100 in PBS containing 5% goat serum for 20 min. Following permeabilization, cells were incubated with galectin-3 antibody (1:100 in 1% BSA, 0.3% Triton X-100 in PBS) overnight at +4°C, washed three times in 0.25% BSA, 0.1% Triton X-100 in PBS and incubated with Alexa Fluor-488 antibody (1:1000 in 1% BSA, 0.3% Triton X-100 in PBS) for 1h at room temperature. Cells were washed three times in 0.05% Tween-20 in PBS and once in cold PBS, mounted using SlowFade Diamond AntifadeMountant with DAPI (ThermoFisher Scientific) and examined by using EVOS FLoidCell Imaging Station (ThermoFisher Scientific).

### Total ROS and superoxide measurements

CM-H_2_DCFDA (ex/em 490/520) and MitoSOX Red (ex/em 510/580) reagents (ThermoFisher Scientific) were used to determine Total ROS and superoxide production, respectively. Briefly, cells were grown on 96-well transparent bottom black plates and stained with CM-H_2_DCFDA or MitoSOX Red according to manufacturer’s protocol. Kinetic measurements of total ROS and superoxide fluorescence levels were processed for 12h every 15 min on Spectramax Gemini microplate fluorometer (Molecular Devices). Data shown are mean±SEM of three independent experiments.

### Z-FRase activity assay (cathepsin)

Cathepsin B/L-specific activity was measured as described before[26]. Briefly, cells were incubated in 210 mM Mannitol, 70 mM sucrose, 20 mM HEPES pH 7.5, 1 mM EDTA, 300 μM Pefabloc, 100 μM PMSF on ice for 45 min followed by homogenization using Dounce homogenizer. The homogenate was centrifuged for 5 min at 350*g* to pellet cellular debris and for 20 min at 16000*g* to obtain mitochondrial fraction. The resulting supernatant was centrifuged at 100000*g* for 45 min to obtain cytosolic extract. 10 μl cytosolic extract was mixed with 85 μl experimental buffer (200 mM sodium acetate, 1 mM EDTA, 0.05% Brij, pH 5.0). 5 μl of z-FR-AMC (25 μM) was added and incubated for 30 min at 37°C. The release of fluorochrome AMC was analyzed at 380 nm excitation and 460 nm emission using Spectramax Gemini microplate fluorometer.

### Statistical analysis

Statistical significance of the results was analyzed using Student’s t-tail test using GraphPad Prism 6.2 software. *P<0.05 and **P<0.01 were considered significant.

## Results and discussion

### BIK is involved in DNA damage-induced apoptosis in HCT-116 cells

**Fig 1:**
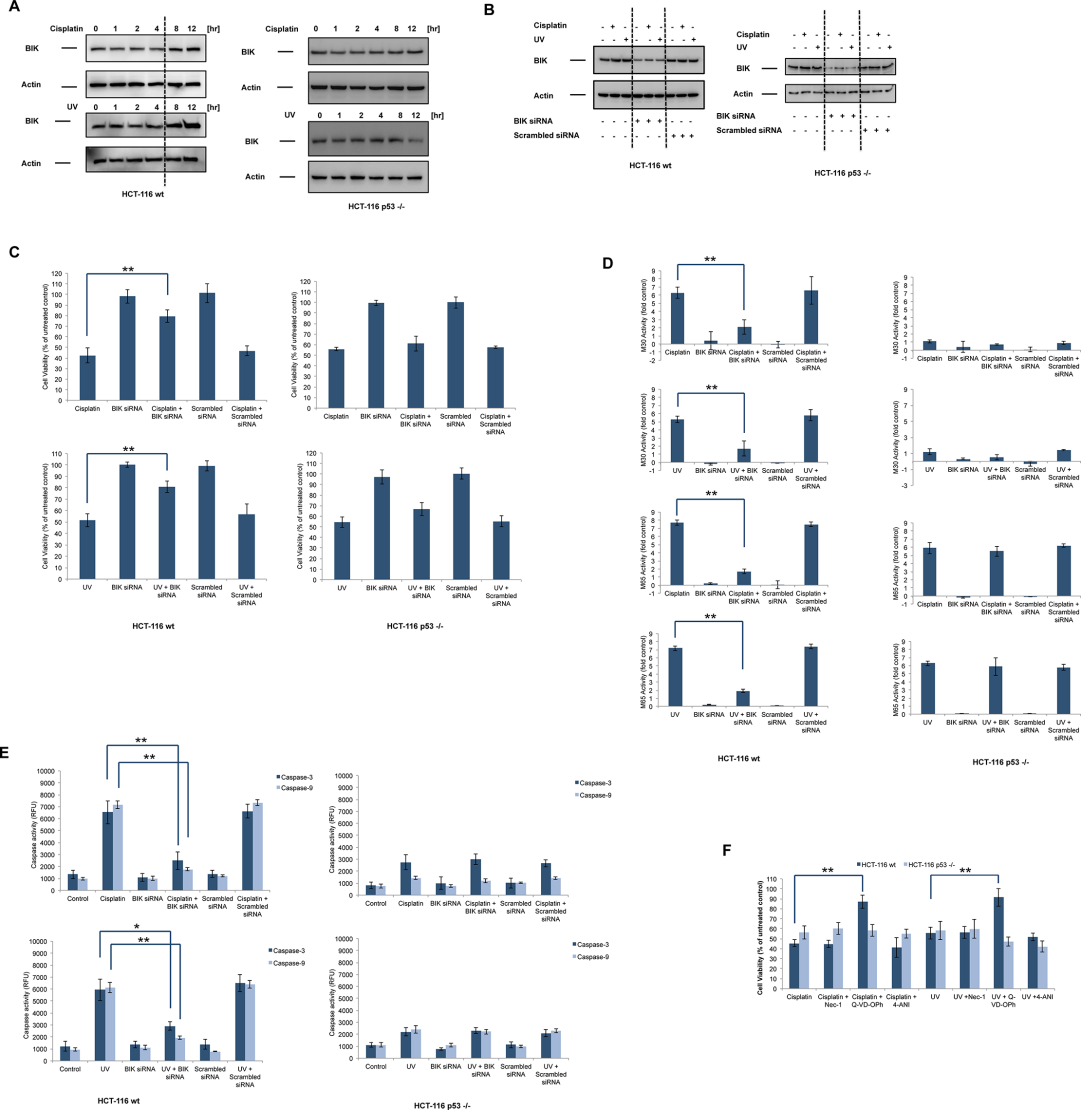
BIK is involved in DNA damage-induced apoptosis in HCT-116 wt cells, but not in HCT-116 p53 -/- cells. (A) HCT-116 wt and HCT-116 p53 -/- cells were treated with cisplatin (20 μM) or UV (100 mJ/cm^2^) for 0-12h and BIK protein levels were detected by immunoblot analysis. Actin was probed as a loading control. (B) HCT-116 wt and HCT-116 p53 -/- cells were transiently transfected with BIK siRNA or scrambled siRNA for 48h. The efficiency of knockdown was monitored by immunoblots. (C) BIK siRNA-transfected or scrambled siRNA-transfected cells were treated with cisplatin (20 μM) or UV (100 mJ/cm^2^) for 48h and cell viability was evaluated by CellTiterGlo assay and expressed as % of untreated control (mean±SEM, n = 3, **P<0.01). (D) BIK siRNA-transfected or scrambled siRNA-transfected cells were treated with cisplatin (20 μM) or UV (100 mJ/cm^2^) for 48h and M30 (apoptosis) and M65 (apoptosis + necrosis) was evaluated by M30/M65 Apoptosense ELISA (mean±SEM, n = 4, **P<0.01). (E) BIK siRNA-transfected or scrambled siRNA-transfected cells were treated with cisplatin (20 μM) or UV (100 mJ/cm^2^) for 24h and caspase-3 and caspase-9 activities were determined by fluorometric caspase assays (mean±SEM, n = 3, *P<0.05, **P<0.01). (F) Cells were pretreated with Nec-1 (20 μM), Q-VD-OPh (20 μM), 4-ANI (2 μM) for 2h and then treated with cisplatin (20 μM) or UV (100 mJ/cm^2^) for 48h. Cell viability was evaluated by CellTiterGlo assay and expressed as % of untreated control (mean±SEM, n = 3, **P<0.01).

Previous reports demonstrated the essential role of BIK in both caspase-dependent and caspase-independent cell death pathways. It has been shown that BIK was largely localized in the endoplasmic reticulum (ER) and BIK-induced release of Ca2+ from the ER in augmented mitochondrial cytochrome c release occurs through Drp-1-mediated remodeling of mitochondrial cristae [5, 6, 31]. The release of calcium from the ER by BIK was dependent on the recruitment of BAX and BAK to the ER. In MCF-7 breast cancer cells, treatment with doxorubicin, γ-irradiation or fulvestrant increased BIK mRNA and protein levels[32].Although direct p53 transcriptional activity was required for doxorubicin- or γ-irradiation-mediated induction of BIK, this was not the case for fulvestrant, in which p53 indirectly regulated BIK levels. Similarly, reconstitution of p53 in p53-null H1299 lung carcinoma cells resulted in increased BIK mRNA and protein levels along with the induction of apoptosis[33]. In contrast, etoposide induced comparable levels of BIK mRNA in Eμ-Myc and Eμ-Myc/p53 -/- lymphoma cell lines, indicating p53-independent induction of BIK[34]. Moreover, loss of BIK did not accelerate Eμ-Myc-induced lymphomagenesis, did not protect Eμ-Myc/BIK -/- lymphoma cells against etoposide or did not affect the response of Eμ-Myc/BIK -/- lymphomas to cyclophosphamide treatment *in vivo*. To test the alteration of BIK levels in response to DNA damage in HCT-116 wt and p53 -/- cells, we treated the cells with (20 μM) or UV (100 mJ/cm^2^) for0-12h. BIK protein levels were detected by immunoblot analysis. As shown in Fig 1A, treatment with cisplatin or UV resulted in increased BIK levels after 8h and 12h in HCT-116 wt cells but not in HCT-116 p53 -/- cells. After confirming the steady-state expression status of p53 in HCT-116 wt and p53 -/- cells, we determined BIK mRNA levels in both cells lines following treatment with cisplatin or UV (S1A). Cisplatin and UV treatment led to increased BIK mRNA levels in HCT-116 wt cells, but not in HCT-116 p53 -/- cells (S1 Fig). Contrary to our results, Ral et al. demonstrated that BIK could be induced by doxorubicin and cisplatin in HCT-116 p53-/- cells through activation of E2F transcription factors[7]. They used a higher dose of cisplatin (30 μM) and incubated cells longer (24h) with the drugs before total RNA isolation, which could explain this discrepancy. Furthermore, we could not detect any difference in BIK protein stability in HCT-116 wt and p53 -/- cells (S1 Fig). Next, we depleted BIK levels in HCT-116 wt and HCT-116 p53 -/- cells by using siRNA duplexes. As seen in Fig 1B, BIK protein levels were efficiently reduced by siRNA treatment and scrambled siRNAs did not have any notable effect. Depletion of BIK significantly decreased cisplatin- or UV-induced cell death in HCT-116wt cells, although no such an effect was observed in HCT-116 p53 -/- cells (Fig 1C). Supporting our results, Real et al. showed that knockdown of BIK by siRNA-mediated silencing protected HCT-116 wt cells against doxorubicin-induced apoptosis, but the response of HCT-116 p53 -/- cells was not assessed[7]. To rule out any effect of BIK knockdown on p53 transcriptional activity, we monitored the transcriptional activation of p53 upon exposure to cisplatin or UV in BIK siRNA-treated HCT-116 wt cells by using luciferase reporter assays. S1 Fig shows that depleting BIK did not have any effect on cisplatin- or UV-induced p53 transcriptional activation. Furthermore, adding BIK BH3 peptide directly to the cytoplasmic extracts used for luciferase reporter assays did not alter p53 transcriptional activation. Therefore, the involvement of BIK in DNA damage-induced cell death in HCT-116 wt cells was not due to the alteration of p53 transcriptional activity.

**Fig 2:**
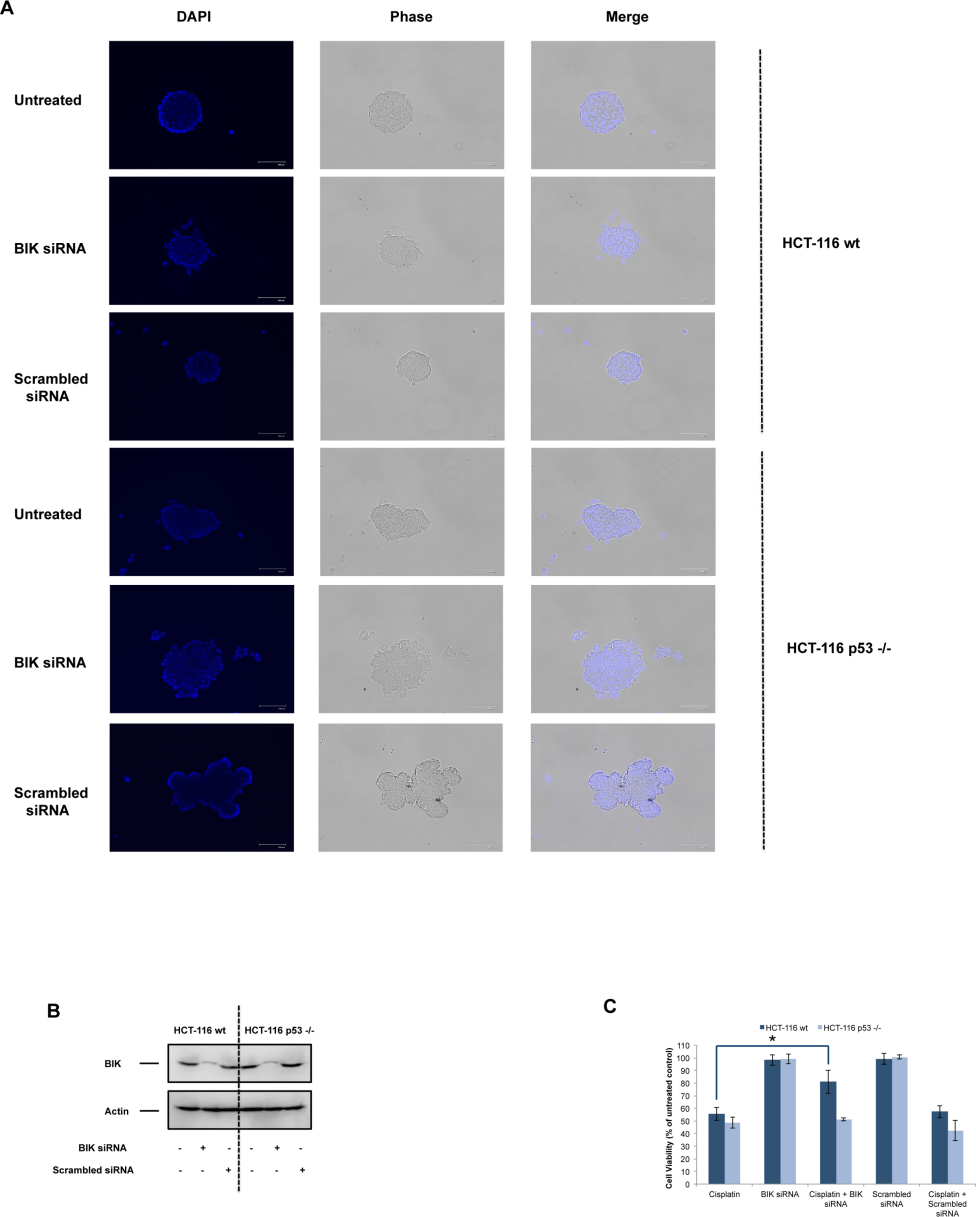
BIK mediates cisplatin-induced cell death in HCT-116 wt cellular spheroids, but not in HCT-116 p53 -/- spheroids. (A) HCT-116 wt and HCT-116 p53 -/- cells were transfected with BIK siRNA or scrambled siRNA for 24h. Cells were subsequently grown in 24-well 3D Algimatrix plates in the presence of RNAi duplexes. Microscopic evaluation of spheroids was done to verify that siRNA treatments did not interfere with the 3D growth of HCT-116 cells. (B) The efficiency of BIK depletion by RNA interference in HCT-116 wt and HCT-116 p53 -/- was determined by immunoblot analysis. Actin was probed as loading control. (C) Spheroids were treated with cisplatin (200 μM) for 48h and cell viability was assessed by using alamarBlue assay (mean±SEM, n = 3, *P<0.05).

DNA damaging agents can induce apoptotic or necrotic cell death in different cell types[35]. Thereby, we evaluated the mode of cell death in HCT-116 wt and p53 -/- cells by using M30 and M65 Apoptosense ELISA assays.M30 ELISA detects the CK18Asp396 epitope after caspase-mediated cleavage and selectively evaluates apoptotic cell death, while M65 ELISA detects full-length CK18 as well as caspase-cleaved fragments, measuring both apoptosis and necrosis[27]. In HCT-116 cells, cisplatin or UV induced both M30 and M65 activity, which were decreased by siRNA-mediated BIK depletion (Fig 1D). In contrast, cisplatin or UV treatment led to increased M65 activity, but not M30 activity in HCT-116 p53 -/- cells and siRNA-mediated BIK depletion did not have any effect on increased M65 activity (Fig 1D). In addition to its involvement in mitochondrial apoptotic signaling, BIK was shown to mediate the caspase-independent cell death response. In SK-Mel-13 cells, doxycycline-induced expression of BIK promoted cell death without caspase activation and mitochondrial cytochrome *c* release[9]. Overexpression of BIK in BCL-2 -/- mouse fibroblasts resulted in cell death without activation of caspase-3 or caspase-9 or mitochondrial cytochrome c release[36]. Intriguingly, an increased cell death response was observed in BIK-expressing BCL-2 -/- cells when cells were treated with pancaspase inhibitor zVAD-fmk. These findings prompted us to examine the role of BIK induction in cisplatin- or UV-induced caspase activation in HCT-116 wt and p53 -/- cells. Treatment of HCT-116 wt cells with cisplatin or UV activated caspase-3 and -9 (Fig 1E) and induced the release of cytochrome c into the cytosol (S1 Fig). Knockdown of BIK significantly decreased cisplatin or UV-induced caspase activation and mitochondrial cytochrome c release as shown by siRNA-mediated depletion assays (Fig 1E and S1 Fig). Cisplatin or UV only minimally activated caspase-3/-9 and partially induced the release of cytochrome c into the cytosolin HCT-116 p53 -/- cells. Of note, decreasing BIK levels by means of RNAi also inhibited this minimal caspase activation response or partial mitochondrial cytochrome c release seen in HCT-116 p53 -/- cells.These results posited that cisplatin and UV trigger the mitochondrial apoptotic pathway in HCT-116 wt cells. On the contrary, cisplatin and UV induces primarily caspase-independent non-apoptotic cell death in HCT-116 p53 -/- cells together with minimal activation of caspases and cytochrome c release without any significant contribution to cell death response. To further support our data that cisplatin and UV preferentially activated mitochondrial apoptosis in HCT-116 wt but not in HCT-116 p53 -/- cells, we determined the activation of BAX and BAK in these cells by flow cytometer. Cisplatin or UV treatment activated BAX and BAK in HCT-116 wt cells which was inhibited by BIK siRNA but not by scrambled siRNA (S2 Fig). We could not detect BAX or BAK activation in response to cisplatin or UV treatment in HCT-116 p53 -/- cells and neither BIK siRNA nor scrambled siRNA had an effect (S3 Fig). To pinpoint the involvement of different cell death pathways activated in HCT-116 wt and p53 -/- cells, we exposed the cells to Nec-1 (inhibitor of necroptosis), Q-VD-OPh (pancaspase inhibitor) and 4-ANI (PARP inhibitor) and determined the cell death response by using a CellTiterGlo assay. As demonstrated in Fig 1F, only Q-VD-OPh exerted a protective effect against cisplatin- or UV-induced cell death in HCT-116 wt cells. We also confirmed the involvement of BIK in cisplatin-induced apoptosis in HCT-116 wt cells grown as 3D spheroids, which may better mimic the *in vivo* milieu than 2D cell culture systems (Fig 2A). Spheroids were transfected with BIK siRNA or scrambled siRNA to deplete BIK. The efficiency of BIK knockdown in spheroids was evaluated by immunoblot analysis (Fig 2B). Notably, BIK siRNA or scrambled siRNA duplexes did not interfere with 3D spheroid formation of HCT-116 cells in Algimatrix 3D culture plate (Fig 2A). Our results demonstrated that BIK depletion significantly decreased cisplatin-induced cell death in HCT-116 wt spheroids (Fig 2C). No significant difference was foundin scrambled siRNA-treated cells.On the contrary, BIK depletion did not affect cisplatin-induced cell death in HCT-116 p53 -/- cells.Collectively, these results suggested that cisplatin and UV triggers caspase-dependent apoptosis, which requires p53-dependent induction of BIK in HCT-116 wt cells.

### DNA damage induces increased BIK/BCL-2 and BIK/BCL-XL interactions in HCT-116 wt cells

**Fig 3:**
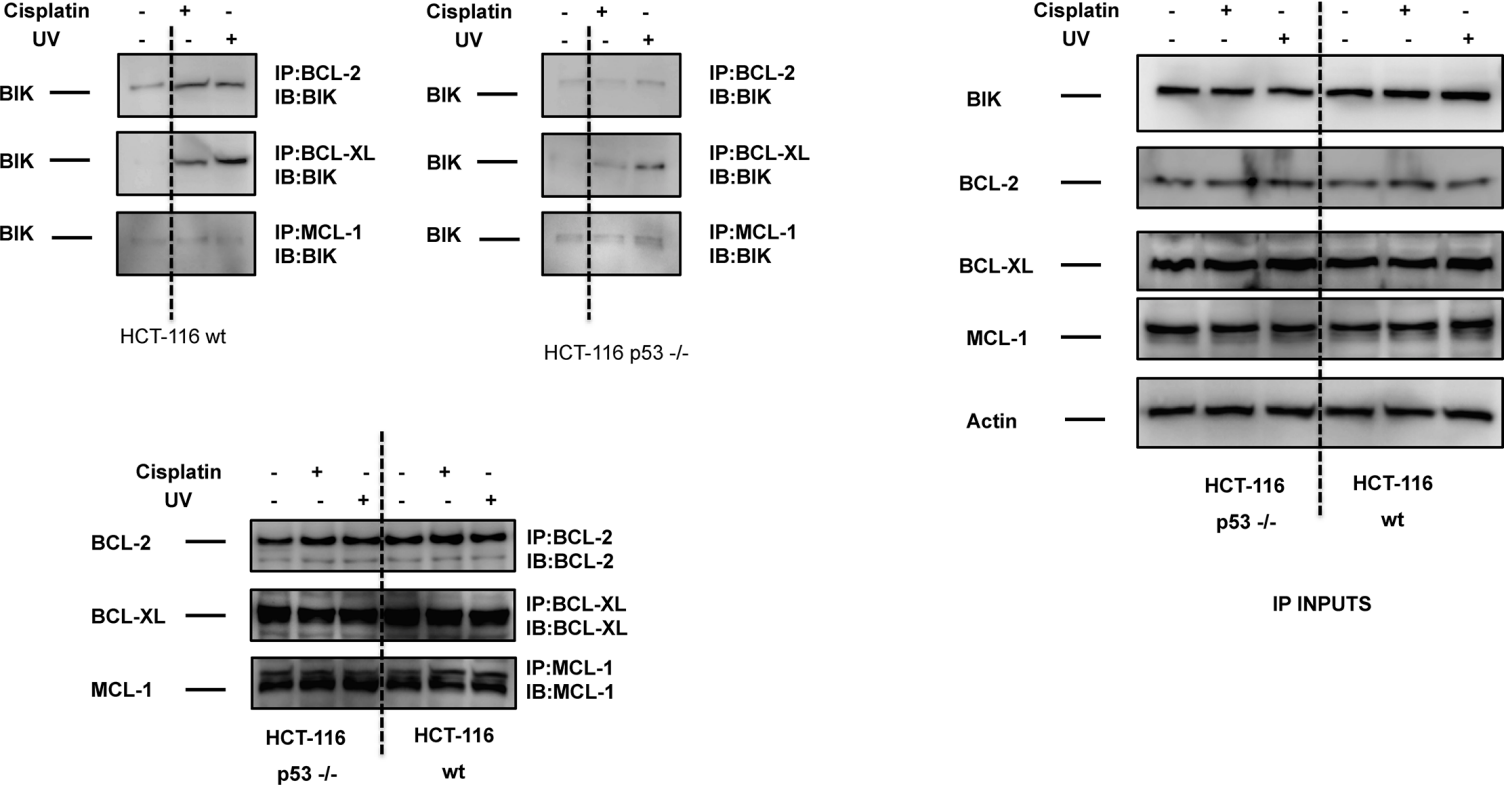
BIK/BCL-2 and BIK/BCL-XL interactions were increased in HCT-116 wt cells in response to DNA damage. HCT-116 wt and HCT-116 p53 -/- cells were treated with cisplatin (20 μM) or UV (100 mJ/cm^2^) for 4h and the interaction of BIK with BCL-2, BCL-XL, and MCL-1 was detected by coimmunoprecipitation assays. Inputs for coimmunoprecipitation experiments were also subjected to immunoblot analysis and actin was probed as loading control.

The dynamic interaction pattern between antiapoptotic and proapoptotic BCL-2 protein family members regulatesmitochondrial outer membrane permeabilization and commitment to cell death in response to various cellular insults[37]. Previous studies showed that BIK could bind and repress antiapoptotic BCL-2 proteins to promote mitochondrial cell death[4, 38–41].To monitor the alteration of protein-protein interactions between BIK and antiapoptotic BCL-2 protein family members (BCL-2, BCL-XL, MCL-1) in response to cisplatin or UV exposure, we took advantage of coimmunoprecipitation experiments in HCT-116 wt and p53 -/- cells. Cisplatin or UV treatment led to increased BIK/BCL-2 and BIK/BCL-XL interactions in HCT-116 cells, but BIK/MCL-1 interaction did not change (Fig 3). In HCT-116 p53 -/- cells, we only detected a slight increase in BIK/BCL-XL interaction; no alteration was observed in BIK/BCL-2 and BIK/MCL-1 interactions (Fig 3). It is possible that other BH3-only proteins are involved in cisplatin- or UV-induced apoptosis in HCT-116 cells, but our results suggest that BIK binds to BCL-2 and BCL-XL to inhibit their prosurvival function following DNA damage. Furthermore, increased BIK/BCL-XL interaction did not trigger a significant apoptotic response in HCT-116 p53 -/- cells following cisplatin or UV exposure, which might be due to either inefficient neutralization of BCL-XL by BIK or residual antiapoptotic buffering provided by BCL-2 and MCL-1.

### Depletion of BIK resulted in decreased mitochondrial apoptotic priming in HCT-116 wt cells

**Fig 4:**
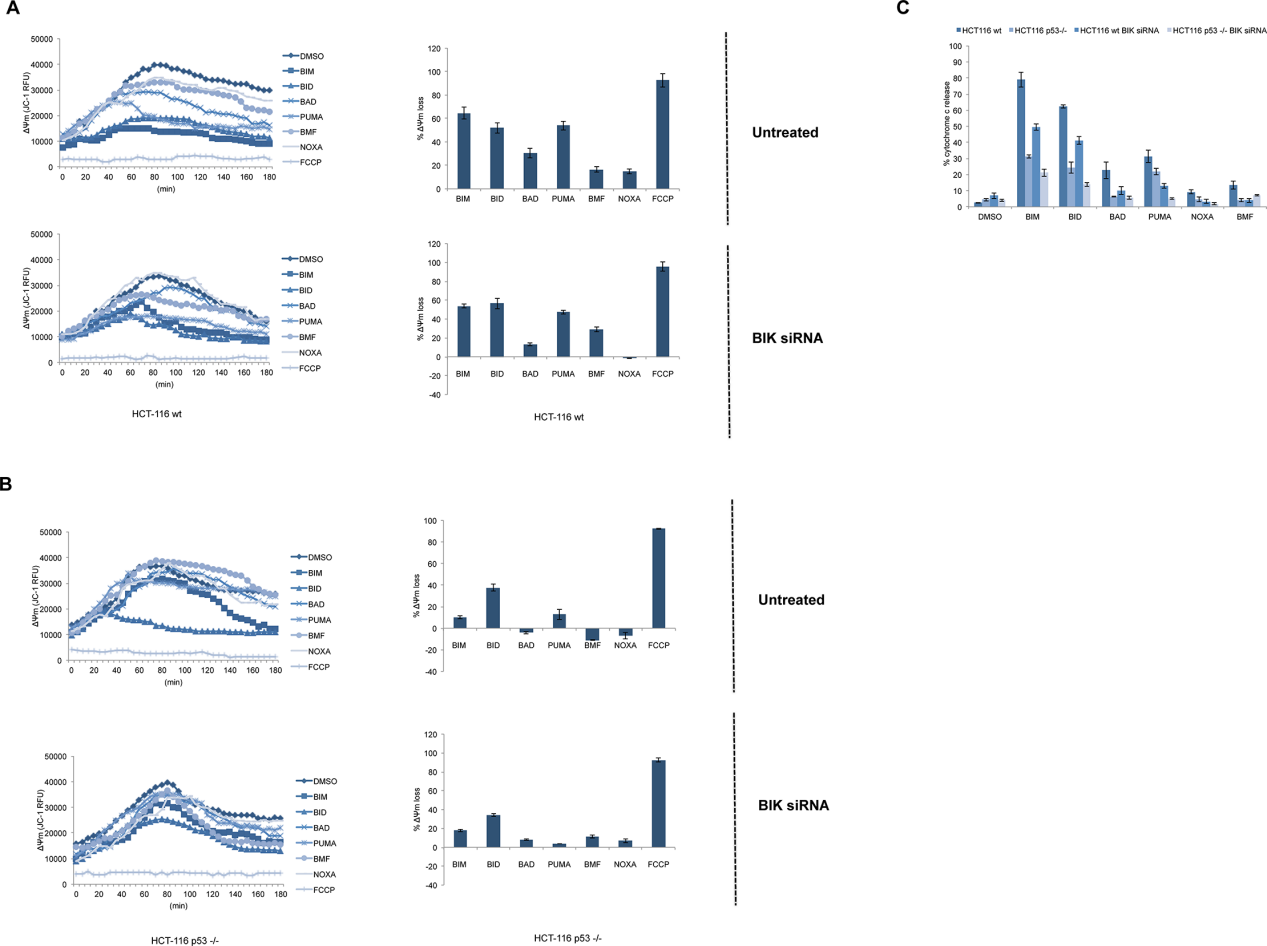
Depletion of BIK led to decreased mitochondrial cell death priming in HCT-116 wt cells. (A) HCT-116 wt and (B) HCT-116 p53 -/- cells were untreated (upper panels) or transiently transfected with BIK siRNA (lower panels) and mitochondrial depolarization was measured following incubation of cells with DMSO, BIM, BID, BAD, PUMA, BMF and NOXA peptides at 100 μM and FCCP at 10 μM. Graphs are shown as mean±SEM, n = 3 and sample mitochondrial potential (ΔΨm) kinetic tracings are provided. (C) Microplate-based BH3 profiles were confirmed by ELISA-based cytochrome c release assays following incubation of isolated mitochondria with DMSO, BIM, BID, BAD, PUMA, BMF and NOXA peptides at 100 μM (mean±SEM, n = 3).

Mitochondrial cell death priming defines how close a cell is to the biochemical and cellular threshold of apoptosis[1]. BH3 profiling assay is based on the incubation of BH3 peptides derived from proapoptotic BH3-only proteins, which selectively bind to antiapoptotic BCL-2 proteins, with purified mitochondria or permeabilized whole cells[42].Increased mitochondrial sensitivity to peptides indicates heightened mitochondrial apoptotic priming and an improved response of the cell to chemotherapy[28, 43]. Thereby, we further examined the effect of BIK depletion on mitochondrial cell death priming status of the cells by using q BH3 profiling assay in HCT-116 wt and p53 -/- cells. HCT-116 wt cells were more primed compared toHCT-116 p53 -/- cells and RNAi-mediated BIK knockdown resulted in decreased priming in HCT-116 wt cells (Fig 4A and 4B). In contrast, depletion of BIK by siRNA treatment did not markedly affect mitochondrial priming in HCT-116 p53 -/- cells except decreasing PUMA BH3 peptide response (Fig 4B). ELISA-based BH3 profiling also confirmed the remarkably decreased mitochondrial cell death priming of HCT-116 wt cells in response to BIK knockdown (Fig 4C).We also observed a decreased response to BIM, BID and PUMA peptides following BIK knockdown in HCT-116 p53 -/- cells with ELISA-based BH3 profiling assays, indicating that BIK also contributes to proapoptotic priming in these cells, although to a lesser extent compared to HCT-116 wt cells. These results posit that expression of p53 confers cells more primed to mitochondrial cell death and occupation of the BH3-binding pockets of antiapoptotic BCL-2 proteins by BIK contributes to this heightened priming status. As shown in Fig 3, BIK was already bound to all three antiapoptotic proteins, which explains BH3 profiling results and other BH3-only proteins in untreated cells could not compensate for the loss of BIK by siRNA-mediated silencing. In accordance with our data, downregulation of p53 by shRNA resulted in decreased mitochondrial apoptotic priming in HCT-116 p21 -/- cells along with diminished response to BH3 mimetic ABT-737[44].

### DNA damage induces biphasic ROS production in HCT-116 wt cells

**Fig 5:**
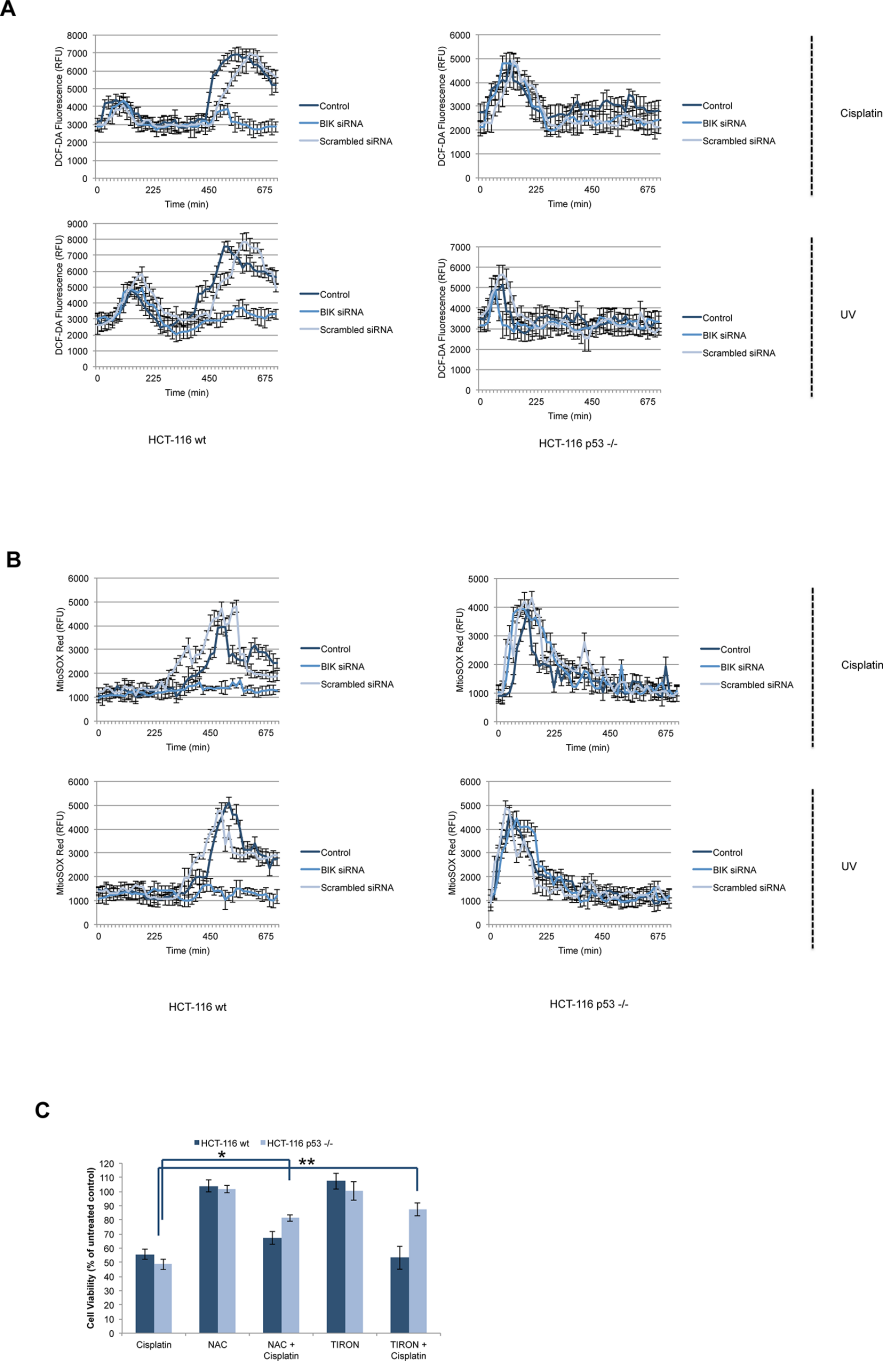
DNA damage induces biphasic ROS production in HCT-116 wt cells. HCT-116 wt and HCT-116 p53 -/- cells were transfected with BIK siRNA or scrambled siRNA for 24h. (A) Untransfected and transfected cells were treated with cisplatin (20 μM) or UV (100 mJ/cm^2^) and total ROS formation was monitored by using DCF-DA probe. Kinetic tracing was processed by measurements in every 15 min for 12h (mean RFU±SEM, n = 3) (B) Untransfected and transfected cells were treated with cisplatin (20 μM) or UV (100 mJ/cm^2^) and superoxide formation was monitored by MitoSOX Red probe. Kinetic tracing was processed by measurements in every 15 min for 12h (mean RFU±SEM, n = 3). (C) Cells were pretreated with NAC (10 mM) or TIRON (10 mM) for 2h and then treated with cisplatin for 48h. Cell viability was evaluated by CellTiterGlo assay and expressed as % of untreated control (mean±SEM, n = 3, *P<0.05, **P<0.01).

Intracellular ROS levels increase in response to DNA damage, which promotes cell death, survival or senescence by utilizing p53-dependent or p53-independent signaling pathways[35, 45]. Accordingly, p53 modulates basal or inducible ROS formation in cell lines and tumor xenograft modelsacting as an antioxidant to control ROS-induced DNA damage and genomic instability[18].Transcriptional activity of p53 was necessary for this function and increased ROS production in p53-deficient mice promoted a cancer-prone phenotype. Besides these findings, loss of p53 expression or function has been shown to decrease H_2_O_2_-induced PARP activation, ATP depletion and necrotic cell death in mouse embryonic fibroblasts, HCT-116 and MCF-7 cells[16]. In KMM-1 human myeloma cells, enforced expression of BIK resulted in increased ROS formation and knockdown of BIK in U266 human myeloma cells resulted in decreased ROS levels[8]. Thus, these studies led us to investigate the crosstalk of ROS, p53 and BIK in response to DNA damage.We initially monitored total ROS production following treatment with cisplatin or UV in HCT-116 wt and p53 -/- cells 0-12h by using a ROS-sensitive fluorescence probe, DCF-DA. In HCT-116 cells, cisplatin or UV treatment induced a biphasic ROS production, with the initial peak at 1h followed by a second peak at 8h (Fig 5A). However, a high amplitude single ROS peakat 1h was present in HCT-116 p53 -/- cells following treatment with cisplatin or UV. Knockdown of BIK in HCT-116 wt cells led to disappearance of the second peak following treatment with cisplatin or UV (Fig 5A), whereas it did not have any effect in HCT-116 p53 -/- cells. Scrambled siRNA duplex did not alter ROS production. Reasoning that superoxide radical predominantly contributes to intracellular ROS generation, we sought to determine mitochondrial superoxide production by using the MitoSOX Red fluorescence probe in HCT-116 wt and p53 -/- cells after treatment with cisplatin or UV. Cisplatin or UV induced superoxide production at 8h post-treatment in HCT-116 wt cells and at 1h post-treatment in HCT-116 p53 -/- cells (Fig 5B). Depletion of BIK inhibited superoxide production in HCT-116 wt cells, but not in HCT-116 p53 -/- cells. These results suggest that DNA damage-induced superoxide production contributes to total ROS production for the second peak in HCT-116 wt cells and BIK upregulation is involved in the formation of this phase of oxidative stress. In fact, superoxide production also contributes to early, strong induction of ROS in HCT-116 p53 -/- cells in response to DNA damage, but BIK was not involved in this process.Consistent with our findings, DNA-damaging agents neocarzinostatin, hydroxyurea and doxorubicin were shown to induce biphasic ROS production in U2OS and HBL100 cells, with an early burst observed within 1-2h followedby a higher second burst within 4-5h post-treatment[46]. Furthermore, NAC-mediated suppression of the second burst significantly abrogated neocarzinostatin-induced cell death. Ectopic expression of FOXO3 in SH-EP and STA-NB15 neuronal cells also resulted in biphasic ROS production. An early peak of ROS was observed at 4h in SH-EP cells and 12h at STA-NB15 cells followed by a secondary stronger peak at 18h in SH-EP and 48h in STA-NB15 cells[47]. BH3-only protein BIM was demonstrated to mediate the FOXO3-induced early ROS peak in both cell lines. Additionally, NAC pretreatment reduced FOXO3-induced early ROS peak, which was shown to be essential for the secondary apoptosis-inducing ROS peak[47]. Furthermore, docosahexaenoic Acid (DHA) was shown to trigger ROS, p53 activation and cell death in multiple cancer cell lines including colon cancer cells, which could be prevented by pretreatment with antioxidants including NAC[48]. To explore the involvement of ROS production in DNA damage-induced cell death in HCT-116 wt and p53 -/- cells, we pretreated the cells with NAC or TIRON followed by treatment with cisplatin or UV and we measured the cell viability by CellTiterGlo cell viability assay. As demonstrated in Fig 5C, the protective effect of NAC and TIRON was limited in HCT-116 wt cells, but both NAC and TIRON effectively inhibited cisplatin- or UV-induced cell death in HCT-116 p53 -/- cells.These results confirmed the critical role of ROS, predominantly the superoxide radical, in DNA damage-induced cell death in HCT-116 p53 -/- cells. We concluded that both early and secondary ROS peaks in HCT-116 wt cells act as secondary amplifiers of the cell death response upon DNA damage rather than primary activators.In NIH/3T3 cells, electromagnetic radiation exposure led to biphasic ROS production at 6h and 48h posttreatment followed by DNA damage and apoptosis[49]. NAC pretreatment inhibited ROSformation, p53 target gene expression, cytochrome c release and caspase-3 activation in NIH/3T3 cells, which confirmed that early ROS burst acted upstream of DNA damage and p53 activation in response to electromagnetic radiation. Interestingly, depletion of intracellular GSH levels by BSO (buthioninesulfoximine) treatment altered the cisplatin-induced apoptotic response to necrotic cell death, mimicking high dose H_2_O_2_ treatment in U937 human monocytes[50]. Remarkably, cisplatin plus BSO-induced necrotic cell death was not accompanied by decreased ATP levels and could not be inhibited by BCL-2 overexpression. Correspondingly, cisplatin was shown to induce caspase-independent necrosis in A549 and H1229 cells treated with z-VAD-fmk or overexpressing BCL-XL[51]. Cisplatin treatment in A549 BCL-XL-overexpressing cells led to ROS production and addition of NAC prevented caspase-independent necrotic cell death in both A549 and H1229 BCL-XL-overexpressing cells. Induction of caspase-independent cell death in p53-null H1299 cells further suggested p53 was dispensable for cisplatin-induced necrosis.

### Cisplatin induces early LMP in HCT-116 p53 -/- cells

**Fig 6:**
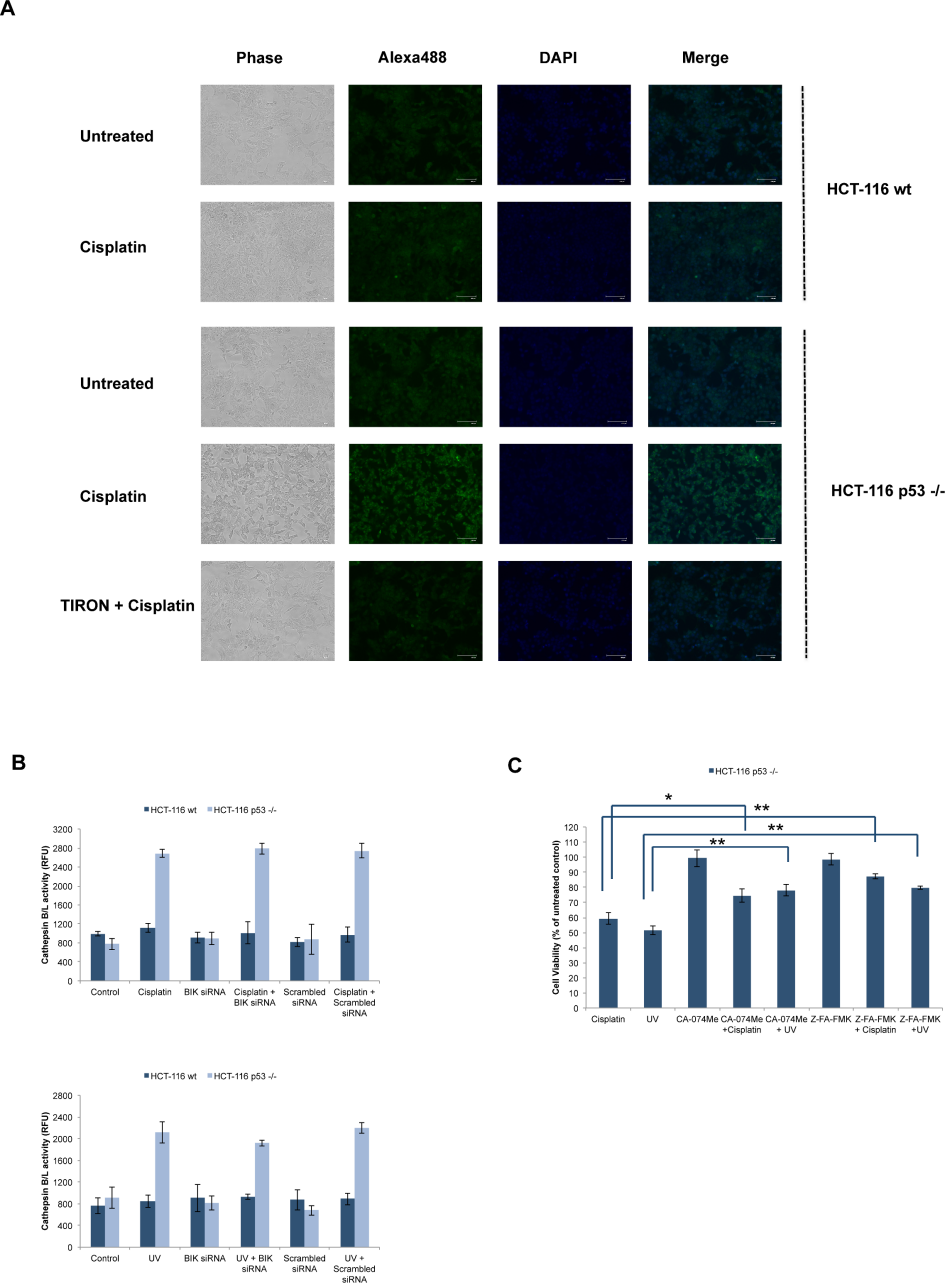
Cisplatin induces early LMP in HCT-116 p53 -/- cells. (A) HCT-116 wt and HCT-116 p53-/- cells were treated with cisplatin (20 μM) for 1h. HCT-116 p53 -/- cells were TIRON (10 mM) for 2h and then treated with cisplatin for 1h to examine the effect of TIRON on cisplatin-induced LMP. Cells were stained for galectin-3 to evaluate the lysosomal membrane permeabilization. (B) HCT-116 wt and HCT-116 p53 -/- cells were transfected with BIK siRNA or scrambled siRNA for 24h. Cells were treated with cisplatin (20 μM) or UV (100 mJ/cm^2^) for 1h and the Cathepsin B/L activity was measured in untransfected and transfected cells (mean RFU±SEM, n = 3). (C) HCT-116 p53 -/- cells were pretreated with CA-074Me (100 μM) or Z-FA-FMK (10 μM) for 2h and then treated with cisplatin (20 μM) or UV (100 mJ/cm^2^) for 48h. Cell viability was determined by CellTiterGlo assay and expressed as % of untreated control (mean±SEM, n = 3, *P<0.05, **P<0.01).

Given the results obtained in HCT-116 p53 -/- cells, we asked whether cathepsins mediated necrosis in response to DNA damage in these cells. It has been found that lysosomal membrane permeabilization and cathepsin activation were involved in necrotic cell death in response to diverse cellular stresses. For example, it was shown that cathepsin C and LMP were necessary for LLOMe-induced lysosome-mediated necrosis, and that cathepsins B and S were involved in RIP-independent, lysosome-mediated necrosis in BALB/c- and C57BL/6-derived macrophages[52, 53].In addition, muramyl dipeptide (MDP) was demonstrated to induce ASC-dependent necrotic cell death in CLC12N2 colorectal cancer cells, which was inhibited by cathepsin B inhibitor CA-074Me, cathepsin L inhibitor IV and cathepsin S inhibitor[54]. Complementing these results, MDP treatment resulted in lysosomal leakage coupled with necrosis as detected by acridine orange staining and flow cytometry analysis.Galectin-3 translocates from the cytosol to lysosomes following lysosomal membrane permeabilization (LMP) and this can be detected by the alteration of immunofluorescence staining patterns from diffuse cytosolic staining to punctate staining[30]. HCT-116 wt and p53 -/- cells were treated with cisplatin for 1h and galectin-3 was detected by using immunofluorescence staining. As shown in Fig 6A, cisplatin induced punctate galectin-3 staining in HCT-116 p53 -/- cells but not in HCT-116 wt cells. Moreover, pretreatment with the superoxide scavenger TIRON prevented cisplatin-induced LMP in HCT-116 p53 -/- cells. Cisplatin or UV also induced cathepsin B/L activation in HCT-116 p53 -/- cells, which was not detected in HCT-116 wt cells (Fig 6B). BIK siRNA or scrambled siRNA did not alter cathepsin B/L activation in HCT-116 wt or p53 -/- cells, indicating BIK-independent LMP in HCT-116 p53-/- cells. Furthermore, cathepsin inhibitors CA-074Me and Z-FA-FMK significantly protected against cisplatin- or UV-induced cell death in HCT-116 p53 -/- cellsconfirming the critical role of cathepsin activation (Fig 6C). Of note, Tu et al. demonstrated that etoposide triggered necrotic cell death in BAX/BAK DKO MEFs and that p53 transcriptional activity, ROS formation as well as cathepsin Q upregulation were necessary for this response[17]. However, lysosomal cathepsins B or L did not have any effect.Moreover, ROS formation in BAX/BAK DKO MEFs did not depend on p53 expression or transcriptional activity and ROS scavengers NAC and DPI protected BAX/BAK DKO MEFs from etoposide-induced necrotic cell death. In addition, they concluded that lysosomal membrane permeabilization occurred secondary to activated necrotic cell death rather than initiating molecular events following DNA damage[17]. In contrast, IL-3deprivation induced LMP and cathepsin B/L activation was completely blocked in BAX/BAK DKO growth factor-dependent monocytes[26]. Additionally, treatment of wt MEFs and monocytes with etoposide or UV resulted in apoptosis, LMP and cathepsin B/L activation secondary to apoptosome activation, which were not seen in BAX/BAK DKO cells. Hence, our results described a different signaling pathway, in which ROS, cathepsin activation and LMP cooperated to trigger p53-independent necrotic cell death. High amplitude ROS formation, mainly composed of superoxide free radical, was the initiating event, which led to LMP and cathepsin activation as shown by the inhibitory effect of TIRON on cisplatin-induced LMP in HCT-116 p53 -/- cells (Fig 6A). Comparable to our results, LMP-inducing compounds were shown to induce p53-independent apoptosis in HCT-116 p53 -/- cells, a shift from caspase-dependent apoptotic cell death to necrosis was observed when higher concentrations of drugs including NSC267461(nanaomycin)[24].

## Conclusions

In conclusion, we identified different modes of cell death induced by DNA damage in HCT-116 wt and p53 -/- cells. DNA-damaging agents can induce apoptosis, necrosis or both depending on the dose and duration, and induction of p53 by drugs does not always indicate p53-dependent apoptosis. We also found that BIK significantly contributes to DNA damage-induced mitochondrial apoptosis in HCT-116 wt cells upstream of the second peak of ROS production, BAX and BAK activation, cytochrome *c* release and caspase activation. On the other hand, an early and high amplitude ROS peak mainly formed by the superoxide radical triggers LMP and cathepsin activation, promoting cisplatin-induced necrotic cell death in HCT-116 p53 -/- cells. Therefore, targeting ROS and LMP as a mechanism of p53-independent cell death in tumors is a promising drug development strategy regarding the mutation rate of p53 in human cancers.

## Supporting information

S1 Fig The crosstalk between BIK and p53 trancriptional activity. (A) p53 expression status in HCT-116 wt and HCT-116 p53 -/- cells were evaluated by immunoblot analysis. HCT-116 wt and HCT-116 p53 -/- cells were treated with cisplatin (20 μM) or UV (100 mJ/cm^2^) for 12h and BIK mRNA levels were detected by real-time qPCR. Results are expressed as fold over control (mean±SEM, n = 3). (B) HCT-116 wt and HCT-116 p53 -/- cells were treated with 10 μg/ml cycloheximide (CHX) and BIK protein levels were detected at 0-24h by immunoblot analysis. Protein expression levels were semi-quantitatively determined by densitometry and shown as a ratio of BIK/Actin. (C) p53 transcriptional activity following treatment with cisplatin (20 μM) or UV (100 mJ/cm^2^) for 12h was measured in untreated or BIK siRNA-treated HCT-116 wt cells using a dual luciferase reporter assay (mean±SEM, n = 3). 100 μM BIK BH3 peptide was added to cell lysates before measurement for Firefly/Renilla dual luciferase activities where indicated. (D) BIK siRNA- or scrambled siRNA-treated HCT-116 wt cells were exposed to cisplatin (20 μM) or UV (100 mJ/cm2) for 16h. The mitochondrial release of cytochrome *c* was evaluated by using an ELISA-based assay (mean±SEM, n = 3, *P<0.05, **P<0.01).
(AI) Click here for additional data file.

S2 Fig Activation of BAX and BAK following BIK knockdown in HCT-116 wt cells. BIK siRNA- or scrambled siRNA-treated HCT-116 wt cells were exposed to cisplatin (20 μM) or UV (100 mJ/cm2) for 12h. Cells were stained for active conformation-specific (A) BAX (6A7) or (B) BAK (Ab-1), followed by incubation with FITC-conjugated secondary antibody. Active BAX-related immunofluorescence was analyzed by flow cytometry. Red, control cells; blue, treated cells.
(AI) Click here for additional data file.

S3 Fig Activation of BAX and BAK following BIK knockdown in HCT-116 p53 -/- cells. BIK siRNA- or scrambled siRNA-treated HCT-116 p53 -/- cells were treated with cisplatin (20 μM) or UV (100 mJ/cm2) for 12h. Cells were stained for active conformation-specific (A) BAX (6A7) or (B) BAK (Ab-1), followed by incubation with FITC-conjugated secondary antibody. Active BAK-related immunofluorescence was analyzed by flow cytometry. Red, control cells; blue, treated cells.
(AI) Click here for additional data file.

S1 File Supplementary materials and methods. Supplementary materials and methods section describing additional experimental procedures including real-time qPCR, assessment of cytochrome *c* release, measurement of BIK protein half-life, p53 reporter assay and flow cytometry-based detection of BAX/BAK activation accompanies this paper.
(PDF)

## Abbreviations

FCCP: Carbonyl cyanide 4-(trifluoromethoxy)phenylhydrazone
TIRON: 4,5-Dihydroxy-1,3-benzenedisulfonic acid disodium salt
NAC: N-Acetyl-L-cysteine)
4-ANI: 4-Amino-1,8-naphthalimide)
Q-VD-OPh: Quinoline-Val-Asp-Difluorophenoxymethylketone
CA-074Me: (L-3-trans-(Propylcarbamyl)oxirane-2-carbonyl)-L-isoleucyl-L-proline methyl ester
