# Supplementary material for: Biphasic ROS production, p53 and BIK dictate the mode of cell death in response to DNA damage in colon cancer cells
Source: PLoS One. 2017 Aug 10;12(8):e0182809. doi: 10.1371/journal.pone.0182809 (PMC5552129; doi:10.1371/journal.pone.0182809)
Supplement: S1 File — Supplementary materials and methods section describing additional experimental procedures including real-time qPCR, assessment of cytochrome c release, measurement of BIK protein half-life, p53 reporter assay and flow cytometry-based detection of BAX/BAK activation accompanies this paper. (PDF) [file pone.0182809.s004.pdf]

Supplementary Materials and Methods

Real-time qPCR

Total RNA was extracted using RNeasy kit (Qiagen). To quantify mRNA expression of BIK, qRT-PCR was carried out using QuantiTect Primer assay (Hs_BIK_1_SG QuantiTect Primer Assay, NM_001197, Qiagen) and 1-step QuantiTect SYBR Green qRT-PCR Kit (Qiagen) according to the manufacturer's standard protocol on LightCycler 480 instrument (Roche). The housekeeping genes GAPDH (Hs_GAPDH_1_SG QuantiTect Primer Assay, NM_002046, Qiagen) was used for normalization and relative gene expression levels were calculated using 2-ΔΔCT method. Results are shown as fold expression over untreated control (mean±SEM, n=3).

Assessment of cytochrome *c* release.

The mitochondrial release of cytochrome c was assayed by using R&D human cytochrome *c* Quantikine ELISA kit (R&D) according to the manufacturer's protocol. Results are expressed as percent cytochrome c release compared with control (mean± SEM, n=4).

Measurement of BIK protein half-life

Cycloheximide (10 µg/ml) was added to HCT-116 wt and HCT-116 p53 -/- cells and cell lysates were isolated at the indicated time points. BIK protein expression was detected by immunoblot and BIK protein expression levels were semi-quantitatively determined by densitometry using ImageJ 1.41 software (ImageJ, NIH, USA), expressed as a ratio of BIK/Actin.

Reporter assay.

p53 reporter assays were performed as described before using Cignal p53 Pathway Reporter Assay Kit (Qiagen)[1]. Briefly, HCT-116 wt cells were transfected with Cignal p53 reporter and luciferase activity was measured using Dual Luciferase Assay system (Promega) on Spectramax Gemini microplate fluorometer. Firefly luciferase experimental reporter was normalized to Renilla luciferase activity to control transfection efficiency. Data were presented as mean± SEM of three independent experiments. 100 µM BIK BH3 peptide was added to cell lysates before measurement for Firefly/Renilla dual luciferase activities as a modification to the protocol where indicated.

BAX and BAK activation by flow cytometry.

Detection of BAX and BAK activation by intracellular staining and flow cytometry using active conformation-specific antibodies anti-BAX (6A7, BD Pharmingen) and anti-BAK (Ab-1, Millipore) was performed as described previously[2]. Activation of BAX or BAK was determined by a shift to the right in the histogram.

References

1. Xie, X. *et al.* Targeting HPV16 E6-p300 interaction reactivates p53 and inhibits the tumorigenicity of HPV-positive head and neck squamous cell carcinoma. *Oncogene* **33**, 1037-1046, doi:10.1038/onc.2013.25 (2014).
2. Panaretakis, T., Pokrovskaja, K., Shoshan, M. C. & Grander, D. Activation of Bak, Bax, and BH3-only proteins in the apoptotic response to doxorubicin. *J Biol Chem* **277**, 44317-44326, doi:10.1074/jbc.M205273200 (2002).
